# I-YOLOv11n: A Lightweight and Efficient Small Target Detection Framework for UAV Aerial Images

**DOI:** 10.3390/s25154857

**Published:** 2025-08-07

**Authors:** Yukai Ma, Caiping Xi, Ting Ma, Han Sun, Huiyang Lu, Xiang Xu, Chen Xu

**Affiliations:** 1 College of Automation, Jiangsu University of Science and Technology, Zhenjiang 212100, China; 231210301212@stu.just.edu.cn (Y.M.); 231210301111@stu.just.edu.cn (T.M.); 221210301114@stu.just.edu.cn (H.L.); 221210301125@stu.just.edu.cn (X.X.); 221210301126@stu.just.edu.cn (C.X.); 2 School of Integrated Circuits, Tsinghua University, Beijing 100084, China; sunhan96@mail.tsinghua.edu.cn; 3 Beijing National Research Center for Information Science and Technology, Beijing 100084, China

**Keywords:** small object detection, YOLOv11n, RFCBAMConv, DFPC, STCMSP, knowledge distillation, anchor clustering, network pruning, UAV

## Abstract

UAV small target detection in urban security, disaster monitoring, agricultural inspection, and other fields faces the challenge of increasing accuracy and real-time requirements. However, existing detection algorithms still have weak small target representation ability, extensive computational resource overhead, and poor deployment adaptability. Therefore, this paper proposes a lightweight algorithm, I-YOLOv11n, based on YOLOv11n, which is systematically improved in terms of both feature enhancement and structure compression. The RFCBAMConv module that combines deformable convolution and channel–spatial attention is designed to adjust the receptive field and strengthen the edge features dynamically. The multiscale pyramid of STCMSP context and the lightweight Transformer–DyHead hybrid detection head are designed by combining the multiscale hole feature pyramid (DFPC), which realizes the cross-scale semantic modeling and adaptive focusing of the target area. A collaborative lightweight strategy is proposed. Firstly, the semantic discrimination ability of the teacher model for small targets is transferred to guide and protect the subsequent compression process by integrating the mixed knowledge distillation of response alignment, feature imitation, and structure maintenance. Secondly, the LAMP–Taylor channel pruning mechanism is used to compress the model redundancy, mainly to protect the key channels sensitive to shallow small targets. Finally, K-means++ anchor frame optimization based on IoU distance is implemented to adapt the feature structure retained after pruning and the scale distribution of small targets of UAV. While significantly reducing the model size (parameter 3.87 M, calculation 14.7 GFLOPs), the detection accuracy of small targets is effectively maintained and improved. Experiments on VisDrone, AI-TOD, and SODA-A datasets show that the mAP@0.5 and mAP@0.5:0.95 of I-YOLOv11n are 7.1% and 4.9% higher than the benchmark model YOLOv11 n, respectively, while maintaining real-time processing capabilities, verifying its comprehensive advantages in accuracy, light weight, and deployment.

## 1. Introduction

With the widespread deployment of unmanned aerial vehicle (UAV) technologies in fields such as urban security surveillance [1], environmental monitoring [2], and disaster emergency response [3], their role in aerial information acquisition and decision-making assistance has become increasingly critical. However, constrained by factors such as operational altitude, illumination variations, viewpoint deviations, and onboard computational resource limitations, the detection of small objects (typically < 32 × 32 pixels) in aerial imagery remains challenging. Key difficulties include complex backgrounds, congested multi-object distribution, blurred object boundaries, and drastic scale variations [4]. Achieving high-accuracy and low-latency small object detection on resource-constrained embedded platforms has emerged as an active research focus in computer vision.

In recent years, deep convolutional neural network (CNN)-based object detection frameworks such as the YOLO series [5,6,7], Faster R-CNN, and SSD have demonstrated remarkable progress in standard scenarios. As a recent advancement in this lineage, YOLOv11n [8] exhibits significant advantages in both speed and lightweight design. However, when deployed for small target detection in UAV aerial imagery, such models exhibit three persistent limitations [9]: (1) Feature degradation: repeated downsampling and convolutional operations cause subtle target features to be obscured by background noise, thereby impeding effective edge and semantic representation. (2) Rigid receptive fields and scale inflexibility: conventional fixed convolutional kernels lack dynamic adaptability to target scale variations, thereby compromising the detection consistency. (3) The scale of the model is limited: although the complex structure can improve detection accuracy, there are performance bottlenecks in deploying mobile devices.

To this end, current research is optimized in the direction of attention mechanism [10], multiscale feature fusion [11], and model compression [12]. For example, YOLOv8 [13] improves the balance between speed and accuracy by enhancing the feature pyramid structure. However, its fixed convolution kernel causes the edge features of shallow small targets to be submerged by the background, and the standard SPPF structure is challenging to adapt to the drastic change of target scale in aerial images. Deformable DETR [14] introduces a deformable self-attention mechanism to improve the accuracy of target positioning, but its complexity is too high. In high-resolution aerial images, the inference delay of Jetson TX2 exceeds 200 ms, which cannot meet the real-time requirements. For the task of small target detection, Li et al. [15] introduced RFCBAMConv and DFPC modules into YOLOv11n to improve the ability of receptive field and context modeling. Although the mAP index is effectively improved, its detection head structure has limited support for multiscale collaborative modeling, and the lightweight strategy lacks a cross-layer knowledge transfer mechanism.

To address the aforementioned issues, this paper proposes a lightweight UAV small target detection algorithm, combining the latest research results with the improved YOLOv11n. It innovates around three main lines: “multiscale enhancement + attention modeling + model compression”. The specific research contributions are as follows:

- We propose a multiscale feature enhancement architecture—RFCBAMConv (Receptive Field and Channel Block Attention Module Convolution). This module seamlessly integrates deformable convolution with channel-spatial co-attention mechanisms to construct an adaptive receptive field structure, thereby significantly enhancing the perception capabilities for the edge and texture characteristics of small targets.
- The fusion structure of dilated feature pyramid convolution (DFPC) and STCMSP features is designed. Combined with the CGBD module and the OmniKernel strategy, multi-level context feature compensation is achieved to enhance the semantic modeling capability in complex backgrounds.
- We redesign the Dynamic Detection Head (DyHead) with a hybrid Transformer mechanism. Building upon DyHead’s dynamic weighting framework, we integrate a cross-scale self-attention mechanism to strengthen spatial–channel–scale triple-feature co-modeling. This significantly enhances the localization consistency and classification accuracy for small targets in complex backgrounds, while boosting the detection robustness and positioning stability.
- We propose a three-stage lightweight strategy, “Distillation–Anchor–Pruning”. This approach employs hybrid knowledge distillation to transfer semantic capabilities from teacher models, refines anchor distribution via K-means++ clustering optimization, and compresses channel redundancy using LAMP–Taylor pruning, achieving an effective balance between accuracy and computational efficiency.
- Experiments are carried out on the three aerial datasets of VisDrone, AI-TOD, and SODA-A. The results show that the proposed algorithm increases by 7.1% and 4.9%, respectively, in mAP@0.5 and mAP@0.5:0.95, and the calculation amount is reduced to 14.7 GFLOPs. The parameter quantity is controlled within 3.87 M, and it can maintain real-time processing capability (24 FPS) on embedded platforms (such as Jetson TX2) to meet the deployment requirements of embedded terminals.

The subsequent organization of this paper is structured as follows: Section 2 surveys the related work, covering fundamental object detection techniques, the YOLOv11n architecture, and lightweight technologies. Section 3 details our improved YOLOv11n framework, which includes enhanced feature extraction mechanisms, an optimized dynamic detection head design, and a three-stage lightweight strategy. Section 4 presents comprehensive experimental setups, results analysis, and comparative evaluations. Section 5 concludes with the research achievements and proposes future research directions.

## 2. Related Work

### 2.1. Research on UAV Small Target Detection Algorithms

**Figure 1:**
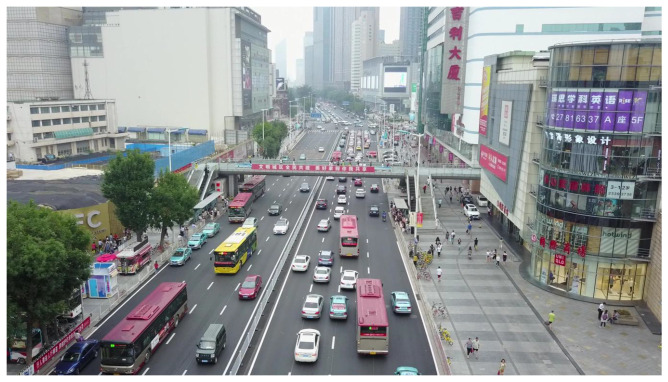
Image from the drone’s point of view.

The detection of small targets in UAV aerial images (as shown in Figure 1) is accompanied by the weakening of target features, complex background interference, and dynamic environmental changes (such as illumination fluctuations and weather effects), which pose dual challenges to the resolution and real-time performance of detection algorithms. Methods based on deep learning are mainly divided into single-stage and two-stage detection frameworks. Among them, single-stage models such as the YOLO series [16] (including YOLOv5 and YOLOv7) and SSD [17] have become the mainstream choice for UAV platforms due to their high efficiency and accuracy. However, the traditional single-stage model still faces three main challenges in detecting small targets with UAVs. First, the feature weakening problem in the standard convolution extraction process makes it challenging to retain the edges and textures of small targets. For example, for YOLOv5, its default pyramid starts from P3, and the anchor frame comes from the general dataset distribution. When faced with UAV micro-targets, the shallow high-resolution features are not directly used for detection, and the edge/texture is weakened in multiple downsamplings. Overlapping the anchor frame and data distribution mismatch and IoU’s excessive sensitivity on micro-targets can easily lead to missed detection and positioning jitter. Second, the fixed receptive field structure limits the model’s ability to adapt to changes in target scale [18]. For example, YOLOv7 introduces E-ELAN, re-parameterization, and trainable freebies in the trunk, neck, and training strategies to make it outstanding on the AP-FPS curve of the general scenario. However, its tiny variant often sacrifices the receptive field and cross-scale representation in pursuit of speed and still uses the anchor frame/IoU as the core mechanism. The context modeling of high-resolution branches is relatively insufficient; so, it has limited robustness to extremely small targets and a high missed detection rate. Third, the model parameters are significant, and the computational complexity is high, which affects the real-time deployment performance. For example, Deformable DETR/RT-DETR avoids the delay and error propagation introduced by NMS through deformable self-attention and end-to-end decoding and shows improvements in small target positioning; however, its decoder layer and multiscale feature fusion bring large computational and memory overhead, which is not friendly to embedded terminals such as Jetson, and the quantitative comparison of open end-to-end reasoning is relatively insufficient, resulting in a lack of real time and deployability on resource-constrained platforms. To enhance the representation of small targets, researchers have focused on multiscale feature fusion and attention mechanisms. Lin et al. pioneered the Feature Pyramid Network (FPN), which enhances hierarchical feature representation through multiscale semantic propagation pathways. Nevertheless, its shallow layers exhibit constrained discriminative power due to insufficient semantic richness in low-level feature representations. In recent years, the attention mechanism has been introduced to enhance the ability of feature expression. The convolutional block attention module (CBAM), constructed by Woo et al., introduces channel and spatial attention mechanisms to strengthen the focus on important feature regions, achieving good results in large networks such as ResNet101. However, CBAM has limited performance improvement on lightweight models such as YOLOv5s and MobileNet and is prone to attention bias problems in complex backgrounds.

Recent studies have explored the use of context information and the Transformer architecture to optimize the performance of UAV scenarios. The Swin Transformer proposed by Liu et al. [19] reduces the amount of global self-attention calculation through local window division and movement mechanisms. It improves the spatial relationship modeling ability in small target scenarios. However, its reasoning speed still falls short of meeting the real-time requirements of the UAV side. To overcome the above problems, Li Bin et al. introduced the RFCBAMConv and DFPC modules into the YOLOv11n model, which effectively improved the model’s feature modeling ability for small targets and enhanced context consistency through the DyHead detection head. However, this method still has room for improvement in terms of lightweight design and target perception consistency in complex scenes, particularly in its generalization ability to handle high-density, small-size, and multi-occlusion targets. In summary, a key problem in the current research is how to achieve a balance between feature-rich expression and structural compression, while satisfying the real-time, robustness, and generalization capabilities of the UAV platform. Therefore, this paper proposes a new UAV small target detection framework that integrates dynamic adjustment of receptive field, context-aware fusion, multiscale feature modeling, and multi-strategy lightweight compression to break through the bottleneck of existing methods in precision–efficiency collaborative optimization.

### 2.2. Lightweight Target Detection Model Design

Aiming at the application scenarios of UAV platforms with limited resources and high deployment costs, lightweight design has become a key direction in research on target detection algorithms. The existing technologies primarily focus on three paths: network compression, knowledge distillation, and anchor mechanism optimization, aiming to reduce computational and storage overhead while maintaining effective detection accuracy.

Early approaches primarily relied on parameter pruning and quantization strategies. Han et al. [20] proposed Deep Compression, a technique that compresses VGG-16 model parameters by up to 49× through a three-stage process of pruning–quantization–encoding, significantly reducing the model size. However, directly transferring this strategy to single-stage small object detectors often results in severe degradation of micro-scale target features, thereby compromising the robustness of downstream localization tasks. To address this limitation, subsequent research introduced structured pruning methods to enhance the model structural stability while preserving critical feature pathways. Lee et al. [21] developed the LAMP (Layer-Adaptive Magnitude-based Pruning) method, which enables automatic threshold adjustment via layer-wise magnitude scoring. Concurrently, Molchanov et al. [22] proposed a gradient-based importance criterion derived from Taylor expansion, simultaneously balancing pruning intensity and model performance recovery. The LAMP method is pruned according to the weight amplitude, and the shallow key channels (such as the P2 layer of 160 × 160 resolution) are mistakenly deleted. The small target feature response is attenuated. Taylor pruning needs to save the gradient information and increase the training memory by 1.8 times.

Concurrently, Knowledge Distillation (KD) has been extensively adopted as an effective technique to enhance the generalization capabilities of lightweight models in object detection tasks. Sun et al. [23] devised a Multi-dimensional Feature Fusion and Distillation (MFFD) mechanism, which transfers hierarchical semantic knowledge from teacher networks to student models, maintaining the detection accuracy while substantially reducing the computational overhead. However, the existing methods focus on high-level semantic transfer, but shallow texture information (such as edges and corners) is not effectively transmitted, and the recall rate of small targets is limited.

In terms of anchor box optimization, the anchor box serves as the initial reference for the regression branch of the detection model, directly affecting the regression accuracy and recall ability of the model. The fixed anchor frame configuration has the problem of mismatching with the data distribution, especially in a UAV aerial image, where the target size is significantly small and unevenly distributed. Wang et al. [6] introduced a training-based adaptive optimization mechanism (termed bag-of-freebies) in YOLOv7, enhancing the detection accuracy while improving the multiscale adaptability. However, their anchor initialization still relies on generic datasets, such as COCO, which compromises generalization in specialized scenarios. Xu et al. [24] proposed a density-aware mechanism that quantifies target density using a Density Index (DI), incorporating density-aware label assignment and feature enhancement strategies to address the challenges of high-density small object detection. Nevertheless, this approach exhibits high deployment complexity on resource-constrained platforms. In contrast, K-means++-based anchor redistribution offers implementation simplicity and strong adaptability. This strategy has been empirically validated for aerial imagery applications across multiple studies [25], demonstrating significant practicality.

Although the above research has made significant progress in the direction of lightweight target detection, the following deficiencies remain: (1): the traditional pruning method can easily destroy the structural stability when dealing with a lightweight detection network, resulting in a significant fluctuation in the accuracy of the model; (2): the existing distillation methods pay more attention to single-scale semantic alignment and lack cross-layer semantic transfer and structure-aware design; (3): anchor box optimization often employs a static clustering strategy, which is challenging to dynamically adapt to the target scale changes of multi-scene and heterogeneous distributions. Aiming to meet the comprehensive requirements of the UAV platform for detection models in terms of accuracy, efficiency, and deployment adaptability, this paper proposes an integrated lightweight optimization strategy embedded in the improved YOLOv11 framework. Specifically, it includes the following: a pruning mechanism based on the structural perception score, fusion of LAMP and Taylor channel importance evaluation strategy, and iterative compression of redundant channel structure to reduce the model complexity; at the same time, the response–feature hybrid knowledge distillation method is introduced, and the unpruned improved YOLOv11s is used as the teacher model. Through the dual mechanism of feature imitation and output alignment, the student model is guided to learn deep semantics and discriminative boundaries, thereby maintaining detection accuracy while compressing the structure. In addition, combined with the K-means++ algorithm based on the IoU distance metric, the anchor box re-clustering of the target box in the training set is performed to dynamically generate an a priori box that is more suitable for the scale distribution of the small target of the UAV, which effectively alleviates the problem of uneven scale. Combined with the above design, the improved model achieves a significant improvement in the detection accuracy of small targets while ensuring real-time performance and deployment adaptability.

## 3. Methods

### 3.1. Network Structure

**Figure 2:**
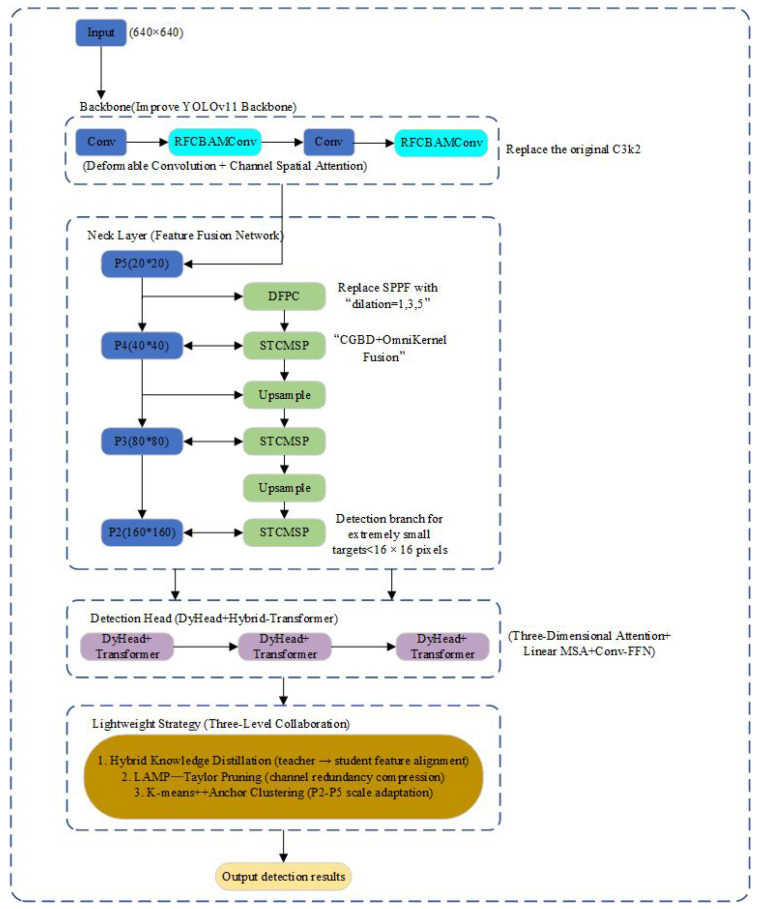
Architecture of the proposed I-YOLOv11n model incorporating RFCBAMConv, DFPC, STCMSP, and DyHead modules.

The lightweight small target detection algorithm for drones proposed in this study, I-YOLOv11n, is based on the YOLOv11n backbone network and incorporates a fusion strategy of “feature enhancement + dynamic detection + three-level lightweighting.” This strategy constructs an end-to-end detection framework integrating perception enhancement, context modeling, anchor box adaptation, and structural compression. The overall structure is shown in Figure 2 and mainly includes the following four key modules:

#### 3.1.1. RFCBAMConv Feature Enhancement Module

To alleviate the problems of weak feature expression and a lack of context information for small targets in complex backgrounds, this paper reconstructs the feature extraction unit based on the original C3k2 structure of YOLOv11n. It introduces the Deformable Convolution [26] and channel–space joint attention mechanism to form the RFCBAMConv module. Firstly, the module adjusts the convolution sampling position through a dynamic offset mechanism to achieve adaptive expansion of the receptive field. Secondly, channel attention based on SE (Squeeze-and-Excitation Networks) structure is used to enhance the expression of key information, and multiscale spatial features are fused by the spatial pyramid pooling (SPP) module to suppress background interference [27]. Experiments demonstrate that the improved RFCBAMConv module effectively enhances the saliency and separability of edge features in small targets, exhibiting stronger detection stability, particularly in complex background scenes.

#### 3.1.2. DFPC Dilated Feature Pyramid Convolution (DFPC) and STCMSP Multiscale Fusion Module

To address the SPPF structure’s insufficient receptive field in YOLOv11n, this paper introduces a multiscale parallel dilated convolution to construct the DFPC module. It utilizes three groups of different expansion rates (1, 3, 5) to construct dilated convolution paths, extracting local, medium-range, and global semantic features in parallel, thereby improving the coverage of the receptive field and avoiding the loss of downsampling information. In addition to enhancing the context modeling ability of small targets, the STCMSP (Small Target Contextual Multiscale Pyramid) module, based on the PAN-FPN structure, is further designed. The module combines the context-guided sampling mechanism [28] and the OmniKernel fusion strategy [29], integrating shallow texture and deep semantic information through a multi-branch structure to effectively enhance the fusion expression of multiscale features and retain local context.

#### 3.1.3. DyHead Detection Head and Hybrid-Transformer Fusion Mechanism

To address the challenges of fuzzy positioning and substantial background interference in detecting small targets, this paper proposes a three-dimensional attention mechanism that combines cross-scale, cross-channel, and cross-space features. It also introduces a lightweight hybrid-Transformer detection head, based on the DyHead structure [30]. The detection accuracy is optimized through scale alignment, adaptive receptive field adjustment, and channel recalibration mechanisms. The hybrid Transformer further combines the linear multi-head self-attention and convolution-enhanced feedforward network (Conv-FFN) module to enhance feature-level semantic modeling and context aggregation capabilities, thereby significantly improving the detection robustness of small targets in complex scenes, such as those with occlusion, variable scale, and multi-background interference. At the same time, the channel activation weights of different target scales are dynamically adjusted through the channel recalibration mechanism, which effectively alleviates the problem of the false detection and missed detection of small targets.

#### 3.1.4. Structure Compression and Anchor Frame Optimization Strategy

In order to realize the efficient deployment of the model on the UAV platform, this paper proposes a three-stage lightweight strategy that integrates knowledge distillation, pruning compression, and anchor frame optimization. Firstly, by constructing a multi-task distillation loss function that integrates response alignment, feature imitation, and structure preservation, the deep semantic information of the uncompressed YOLOv11n teacher model is effectively transferred to the lightweight student model, thereby improving its discriminative ability and generalization performance. Secondly, the importance of the channel is evaluated by combining LAMP and Taylor scoring mechanisms, and the pruning process is guided to maintain the integrity of the key semantic path, especially to retain the modeling ability of the shallow features of P2, thereby avoiding accuracy loss. Finally, the K-means++ anchor clustering algorithm based on IoU distance is used to dynamically generate nine groups of anchor frames to adapt to the size distribution of small targets, focusing on enhancing the perception ability of 160 × 160 resolution branches to tiny targets (<16 × 16 pixels). The above multi-strategy collaborative optimization not only effectively compresses the model scale and calculation amount but also improves the model’s detection accuracy and adaptability to small targets, laying a foundation for subsequent experiments on multiple datasets to verify its deployment value and robustness.

In order to enhance the perceptibility and robustness of small targets in UAV scenes, this paper constructs a collaborative mechanism of ‘alignment–selection–reflux’ in the complete link of ‘backbone–neck–detection head’: DFPC expands a shallow receptive field, RFCBAMConv performs geometric and saliency alignment, STCMSP completes cross-scale context fusion and alignment, DyHead performs adaptive selection with three-dimensional attention and returns the scale selection results to the neck for recalibration through DyGate, to suppress the noise gain and improve the positioning and recall in low light, strong occlusion, and complex background.

### 3.2. Improved RFCBAMConv Module

**Figure 3:**
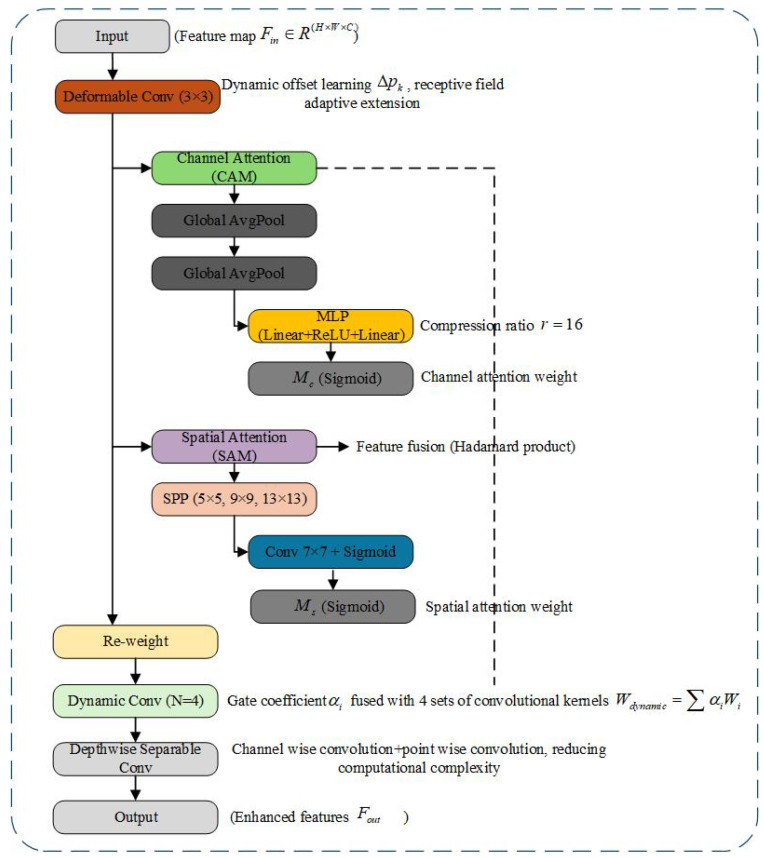
Improved RFCBAMConv model architecture diagram.

Aiming to address the challenges of complex shapes, dramatic scale changes, and significant background interference from small targets from the perspective of a UAV, this paper designs a receptive field and attention-enhanced convolution module with a multi-branch fusion structure, namely the improved RFCBAMConv (Receptive Field and Channel-Block Attention Module Convolution). Based on the RFCBAM structure proposed by Li et al., this module introduces deformable convolution, a hybrid attention mechanism, and a dynamic convolution strategy to comprehensively enhance the robust feature extraction ability of small targets in multiscale and unstructured scenes. Its structure is shown in Figure 3.

#### 3.2.1. Dynamic Receptive Field Modeling: Introducing Deformable Convolution

Due to the fixed receptive field of traditional convolution kernels, they struggle to adapt to the dynamic variations in target shapes and scales within drone imagery. Inspired by the deformable convolution concept proposed by Dai et al., the first layer of our approach employs a deformable convolution mechanism to dynamically adjust sampling positions, thereby achieving receptive field reconstruction based on target features. Specifically, given the input characteristic graph $F_{in}\in R^{H\times W\times C}$, deformable convolution adjusts the sampling position by learning the offset $\Delta p_{k}\in R^{H\times W\times 2K}$, whose output is calculated as

(1) $$y\left(p\right)=\sum_{k=1}^{K}w_{k}\cdot x\left(p+p_{k}+\Delta p_{k}\right),$$

where *p* is the current pixel position, $p_{k}$ is the predefined offset of the standard convolution, and $\Delta p_{k}$ is the learnable dynamic offset. This mechanism enables the convolution kernel to flexibly focus on the texture edge region and improve the boundary modeling ability of blurred small targets.

#### 3.2.2. Hybrid Attention Mechanism (HAM)

To further enhance the modeling ability of key information and eliminate redundant features, this paper proposes a hybrid attention mechanism (HAM) that combines channel and spatial attention. The mechanism draws on the CBAM and SE-Net structures, combining channel attention (CAM) and spatial attention (SAM). The CAM obtains statistical features through global average pooling (GAP) and maximum pooling (GMP). The attention weight $M_{c}$ is calculated by the shared two-layer MLP (weights $W_{0}$, $W_{1}$):

(2) $$M_{c}=\sigma \left(W_{1}\left(W_{0}\left(GAP(F)\right)+W_{0}\left(GMP(F)\right)\right)\right),$$

where $W_{0}$ and $W_{1}$ are the weight matrices sharing two layers of MLP in the channel attention module (CAM). GAP(F) is the feature vector after global average pooling, and GMP(F) is the feature after local maximum pooling. $W_{0}$ performs dimension reduction and feature transformation on GAP(F) and GMP(F), respectively, and $W_{1}$ raises the above fused features back to the original number of channels.

The compression ratio is set to r=16, and σ is the Sigmoid activation function, which is used to highlight the important channel response.The spatial attention module (SAM) utilizes a spatial pyramid pooling (SPP) approach to extract contextual information at various scales. The feature map undergoes max-pooling with kernel sizes of 5 × 5, 9 × 9, and 13 × 13, respectively. These pooled features are then concatenated and processed through a 7 × 7 convolution to generate the spatial attention map $M_{s}$:

(3) $$M_{s}={Conv}_{7\times 7}\left(\left[F;SPP_{5}\left(F\right);SPP_{9}\left(F\right);SPP_{13}\left(F\right)\right]\right).$$

Finally, the feature calibration is completed by fusing the attention results of the Hadamard product:

(4) $$F_{\mathrm{out}}=M_{c}⊙M_{s}⊙F_{\mathrm{in}}.$$

The Hadamard product (⊙) is the multiplication of two matrices of the same dimension by elements, and the dimension remains unchanged after the operation. This mechanism effectively enhances the model’s ability to focus on the salient region of small targets and improves the detection accuracy in complex backgrounds.

#### 3.2.3. Efficient Feature Refinement Strategy: Dynamic Convolution and Deep Separable Convolution

On the basis of attention enhancement, this paper introduces depthwise separable convolution (DSC) to reduce the computational complexity and uses the dynamic convolution mechanism [31] to improve the adaptability. DSC decomposes the standard convolution into channel-by-channel convolution and point-by-point convolution, which significantly reduces the amount of calculation. Dynamic convolution constructs adaptive convolution by introducing a linear learnable gating coefficient $\alpha_{i}$ and fusing N=4 groups of convolution kernel weights $W_{i}$:

(5) $$W_{\mathrm{dynamic}}=\sum_{i=1}^{N}\alpha_{i}W_{i}.$$

Among them, $\alpha_{i}$ is generated by a lightweight gated network. Only about 0.03 M parameter overhead is introduced, and the convolution response can be flexibly adjusted according to the scene features to improve the feature expression ability.

#### 3.2.4. Dynamic Sampling and Attention Weight Coupling Mechanism

The offset learning of deformable convolution provides spatial prior for the attention mechanism. Assuming that the input feature map $F_{\in }R^{H\times W\times C}$, the offset field of the deformable convolution output is Δp. The channel–spatial attention weights interact with Δp in the following ways:

(6) $$M_{s}^{\prime }=G\left(M_{s}⊕\mathrm{Resample}\left(\Delta p\right)\right),$$

where $G\left(\cdot \right)$ is the gating function (Sigmoid), and ⊕ is the sum of elements. This operation makes the spatial attention focus on the deformed region (such as the edge of the small target) and suppresses the background noise. Compared with the traditional CBAM, this design explicitly correlates the target deformation and attention distribution to improve the separability of small target features.

The enhanced RFCBAMConv module significantly boosts the small target modeling capability of the YOLOv11n model in complex backgrounds and multiscale scenarios through receptive field restructuring, hybrid attention calibration, and dynamic convolution optimization. As a critical component of the feature extraction backbone, this module provides high-quality semantic feature support for subsequent DFPC multiscale aggregation and DyHead dynamic detection.

### 3.3. Improved Expansion Feature Pyramid Convolution (DFPC) Module

**Figure 4:**
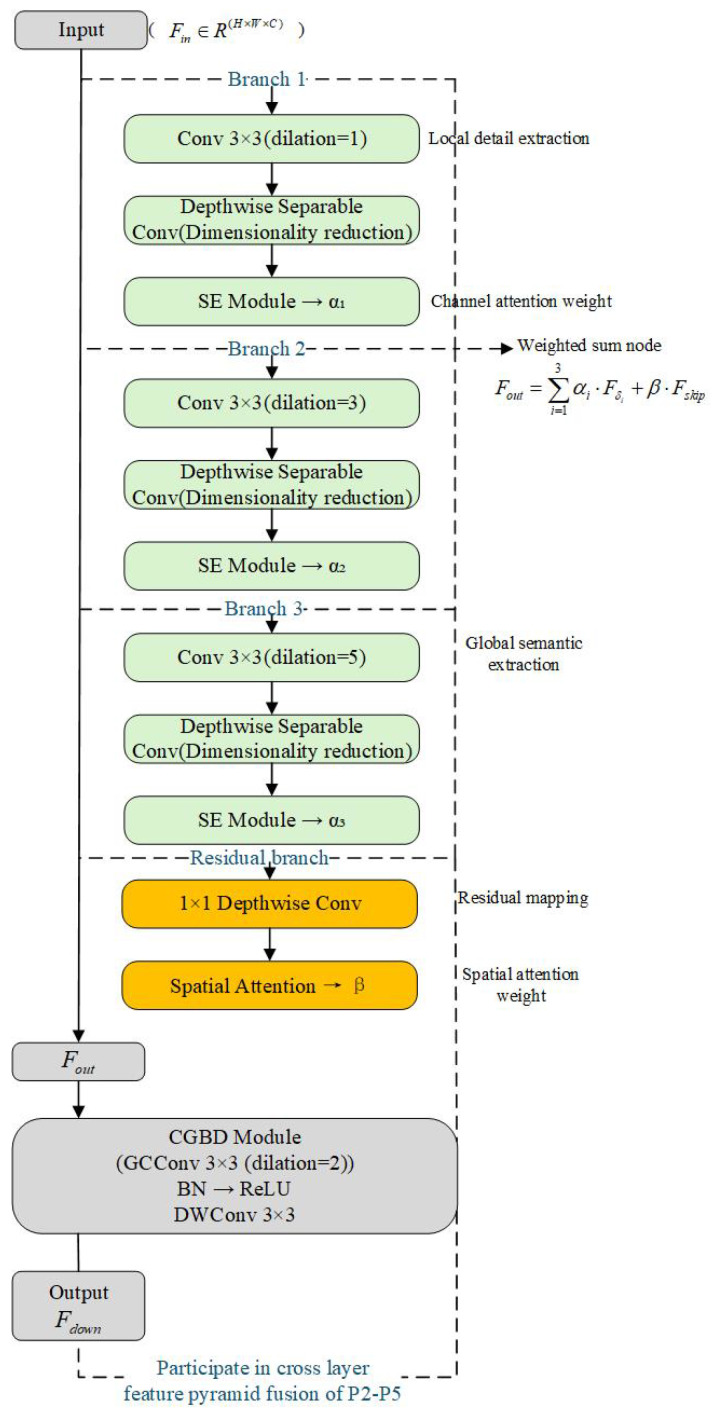
Structure diagram of the improved dilated feature pyramid convolution (DFPC) model.

Aiming at the common bottlenecks of detail loss, receptive field limitation, and static feature fusion in small target detection from the perspective of a UAV, this paper designs an improved dilated feature pyramid convolution (DFPC) module (as shown in Figure 4). The core goal of this module is to explicitly expand the effective receptive field of the model and integrate multiscale context information by parallel multi-branch dilated convolution on the single-layer feature map of the backbone network. It replaces the SPPF layer in the original YOLOv11n. It aims to solve the problem that a single fixed receptive field cannot effectively capture the dramatic changes in the scale of small targets in UAV images and the lack of context information. This module includes the following three key improvements.

#### 3.3.1. Hierarchical Multiscale Dilated Convolution Group Design

To enhance the model’s perception of multiscale small targets and their contextual semantics, this paper designs a DFPC module based on hierarchical multiscale dilated convolution. The module constructs three parallel dilated convolutional branches with dilation rates of 1, 3, and 5, corresponding to the extraction of local details, medium-range structures, and global semantic information, respectively. Specifically, the input feature map $F_{\mathrm{in}}\in R^{H\times W\times C}$ is processed by three sets of 3 × 3 dilated convolution kernels, which are expressed as

(7) $$F_{\delta }={\mathrm{Conv}}_{3\times 3}^{\mathrm{dilate}=\delta }\left(F_{\mathrm{in}}\right),\;\delta \in \left\{1,3,5\right\}.$$

Subsequently, the three features are reduced by depthwise separable convolution and spliced on the channel dimension. Then, the number of channels is compressed by a 1 × 1 convolution to control the amount of calculation, and the fusion output is obtained:

(8) $$F_{\mathrm{concat}}={\mathrm{Conv}}_{1\times 1}\left(\mathrm{Concat}(F_{1},F_{3},F_{5})\right).$$

Among them, $F_{1}$, $F_{3}$, and $F_{5}$ are three-way features. $\mathrm{Concat}(F_{1},F_{3},F_{5})$ represents the stitching of these three feature maps on the channel dimension. ${\mathrm{Conv}}_{1\times 1}$ represents a 1 × 1 convolution operation, which is used to compress the number of channels and control the amount of calculation.

The structure significantly expands the effective receptive field, enabling the collaborative perception of local edge extraction, spatial structure modeling, and global semantic capture, and effectively improves the model’s small target detection performance in complex multiscale scenes.

#### 3.3.2. Dynamic Weight Fusion Mechanism

To fully exploit the relative importance of multi-branch features, this study draws inspiration from the OmniKernel dynamic fusion concept [32]. We introduce channel attention weights $\alpha_{i}$ based on the SE module and a cross-branch residual connection term $F_{\mathrm{skip}}$, achieving adaptive multiscale feature combination through weighted summation:

(9) $$F_{\mathrm{out}}=\sum_{i=1}^{3}\alpha_{i}\cdot F_{\delta_{i}}+\beta \cdot F_{\mathrm{skip}}.$$

Here, $F_{\delta_{i}}$ denotes the output of the *i*-th dilated branch with dilation rate δ, while $F_{\mathrm{skip}}$ represents a cross-branch residual term with dimensionality identical to the input. The weights $\alpha_{i}$ and β are dynamically generated by the SE module (via channel compression and Sigmoid activation) and the spatial attention module, respectively. This mechanism adaptively modulates the contribution of each branch, enhancing feature representation specificity while suppressing redundancy.

(10) $$\alpha_{i}=\sigma \left(W_{2}\cdot \mathrm{ReLU}\left(W_{1}\cdot \mathrm{GAP}\left(F_{\delta_{i}}\right)\right)\right)$$

The residual term $F_{\mathrm{skip}}$ is obtained by 1 × 1 depthwise separable convolution from the input direct mapping. After fusion, the $\beta =\mathrm{Sigmoid}({\mathrm{Conv}}_{7\times 7}\left(\left[\cdot \right]\right))$ generated by the spatial attention mechanism controls its influence intensity.

#### 3.3.3. Lightweight Context-Guided Sampling Alternative Pooling Operation

Traditional maximum pooling has difficulty retaining enough context semantics. This paper introduces the lightweight context-guided sampling module ContextGuidedBlock-Down (CGBD) as an efficient alternative. This module uses global context convolution (GCConv), hole convolution, and batch normalization to preserve context semantics and reduce computing costs. The following is the output formula of the down sampling module:

(11) $$F_{\mathrm{down}}=CGBD\left(F_{\mathrm{out}}\right)=DWConv_{3\times 3}\left(\mathrm{ReLU}\left(\mathrm{BN}\left(GCConv(F_{\mathrm{out}})\right)\right)\right).$$

Here, $F_{\mathrm{down}}$ represents the feature map after downsampling, $F_{\mathrm{out}}$ represents the feature map input to the CGBD module, $DWConv_{3\times 3}$ is a 3 × 3 deep convolution, ReLU is a modified linear unit activation function, and BN is batch normalization. GCConv is a global context convolution with hole convolution, combined with channel attention $M_{c}$ to guide information sampling:

(12) $$GCConv\left(F\right)={\mathrm{Conv}}_{3\times 3}\left(\mathrm{Dilated}=2\right)\left(F⊗M_{c}\right).$$

Here, $M_{c}$ is the weight vector representing the attention of the channel, and $F⊗M_{c}$ is the attention weight of each channel of *F* multiplied by the corresponding attention weight. Therefore, the operation here is actually channel weighting, that is, scaling each channel through a vector $M_{c}$. ${\mathrm{Conv}}_{3\times 3}\;\left(\mathrm{Dilated}=2\right)$ represents a 3 × 3 dilated convolution with a void ratio of 2. $M_{c}\in R^{C}$ is the channel attention weight generated by the SE module. This module effectively improves the ability to retain the context information of small targets in the downsampling process while maintaining low computational complexity.

Let the size of the input feature map be H × W × C, and the three-way expansion convolution (expansion rate d = 1, 3, 5) of the DFPC module is calculated as follows:

(13) $${\mathrm{FLOPs}}_{d}=H\times W\times K^{2}\times C_{\mathrm{in}}\times n\times C_{\mathrm{out}}/\mathrm{group}.$$

K=3 is the size of the convolution kernel, and the number of groups is $group=C_{\mathrm{in}}$ (deep separable convolution). The total amount of three-way parallel computing is $\sum_{d\in \{1,3,5\}}{\mathrm{FLOPs}}_{d}$. Compared with the standard 3 × 3 convolution, the calculation amount of DFPC only increases by about 18.7%, and the deep separable convolution significantly reduces the number of parameters.

#### 3.3.4. Cross-Layer Multiscale Feature Pyramid Path Integration

In order to further improve the context information aggregation ability of dense small targets at different scales, the core goal of the STCMSP (small target contextual multiscale pyramid) module is to construct feature fusion paths at different levels across the backbone network, to realize the efficient fusion and transmission of shallow high-resolution detail information and deep strong semantic information. It aims to solve the problem of the severe loss of small target information in deep features and inconsistent semantics of cross-level features. In this paper, the DFPC and STCMSP modules work together to significantly enhance the feature transfer and semantic consistency between the backbone network and the detection head.

(14) $$F_{\mathrm{pyramid}}=\mathrm{Upsample}\left(P5\right)⊕P4⊕CGBD\left(P3\right)⊕DFPC\left(P2\right)$$

Through upsampling and downsampling (CGBD, DFPC) operations, all input tensors are adjusted to the same spatial size, corresponding to the resolution of the P4 layer and the same number of channels. The ⊕ in the formula represents the addition of elements. Multiple tensors of the same shape are directly added to the elements of the corresponding position (the same spatial coordinates and the same channel). In the process of multiscale path fusion, this paper uses element-by-element addition (⊕) and bilinear interpolation upsampling to align the spatial resolution of different levels of feature maps (P5 to P2), and introduces (ContextGuidedBlock-Down, CGBD) module instead of traditional pooling to retain local and global context information in the downsampling process. In order to improve the perception ability of small targets in dense scenes, the STCMSP module based on PAN-FPN is further introduced into the fusion path. Combined with context-guided dilated convolution and the OmniKernel multi-branching strategy, the dynamic fusion of local and global features at multiple scales is realized, which effectively enhances the identification of small targets and suppresses background interference.

### 3.4. DyHead Dynamic Detection Head Module

**Figure 5:**
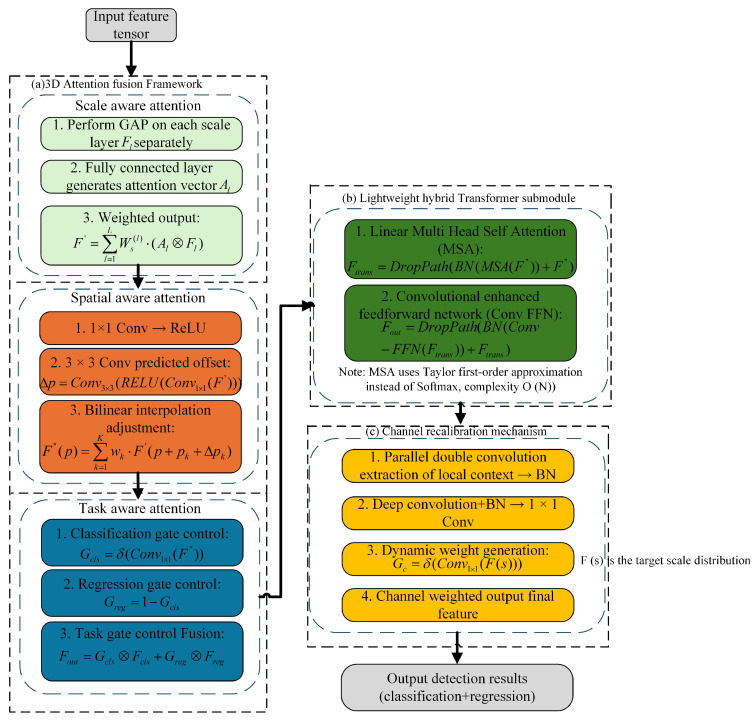
Dyhead model structure.

To address the prevalent challenges in small object detection from a UAV perspective—including localization ambiguity, scale misalignment, and background interference—this paper proposes a hybrid Transformer-enhanced dynamic detection head module based on the DyHead architecture. By integrating a triple-attention mechanism that encompasses scale awareness, spatial awareness, and task awareness, the module achieves cross-layer and cross-task modeling for small objects, thereby significantly improving the discriminative accuracy. The overall structure (Figure 5) comprises three components: a 3D attention fusion framework, a lightweight hybrid Transformer submodule, and a channel recalibration mechanism.

#### 3.4.1. Three-Dimensional Attention Mechanism Framework

The improved DyHead module takes the feature tensor $F\in R^{L\times S\times C}$ (*L* represents the scale dimension, *S* represents the spatial dimension, and *C* represents the channel dimension) output by the YOLOv11n backbone network as input and introduces three types of attention mechanisms in turn:

(1) Scale-aware Attention: To adapt to the drastic scale variations of small objects, we introduce a channel-weighted scale attention mechanism. For each scale layer $F_{l}$, a global average pooling (GAP) operation followed by fully connected layers constructs an attention vector $A_{l}$. The weighted output is given by

(15) $$F^{\prime }=\sum_{l=1}^{L}W_{s}^{\left(l\right)}\cdot \left(A_{l}⊙F_{l}\right).$$

Scale-aware dynamically emphasizes the feature importance of some scale layers through channel-weighted scales and generates $F^{\prime }$. This mechanism guides the model to focus on a more discriminative scale layer, which effectively alleviates the problem of missed detection of small targets due to size differences.

(2) Spatial-aware Attention: To address the issue of false detection resulting from complex background interference, a deformable spatial attention mechanism is proposed. Inspired by the deformable convolution of Dai et al., a deformable convolution enhanced Transformer (deformable Transformer) is introduced to construct a spatial offset prediction network:

(16) $$\Delta p={\mathrm{Conv}}_{3\times 3}\left(\mathrm{ReLU}\left({\mathrm{Conv}}_{1\times 1}\left(F^{\prime }\right)\right)\right),$$

where $\Delta p\in R^{S\times 2K}$ is the predicted spatial offset (K=9 corresponds to 3 × 3 convolution kernel). The feature map $F^{\prime }$, which is dynamically optimized and weighted by Scale-aware, is directly used as the input into the Spatial-aware module. The spatial adaptive adjustment of the feature map is realized by bilinear interpolation:

(17) $$F^{\prime \prime }\left(p\right)=\sum_{k=1}^{K}w_{k}\cdot F^{\prime }\left(p+p_{k}+\Delta p_{k}\right),$$

However, it is worth noting that there is no direct weight multiplication relationship between scale weight and spatial offset, only indirect coupling through feature $F^{\prime }$. This cascade design enables spatial perception to perform more accurate positioning based on scale-optimized features. The two work together to achieve dynamic interactive modeling of scale and spatial information without additional explicit weighting parameters.

(3) Task-aware Attention: To resolve the feature requirement conflict between classification and regression tasks [33], we construct a dual-branch adaptive gating mechanism:

(18) $$\left\{\begin{array}{c} G_{\mathrm{cls}}=\sigma \left({\mathrm{Conv}}_{1\times 1}\left(F^{\prime \prime }\right)\right)\\ G_{\mathrm{reg}}=1-G_{\mathrm{cls}} \end{array}\right..$$

Here, σ is the Sigmoid function, and $G_{\mathrm{cls}}\in {\left[0,1\right]}^{S\times C}$ controls the activation degree of classification features. The final output is fused by task gating:

(19) $$F_{\mathrm{out}}=G_{\mathrm{cls}}⊙F_{\mathrm{cls}}+G_{\mathrm{reg}}⊙F_{\mathrm{reg}}.$$

The strategy alleviates the task optimization conflict and improves the positioning consistency.

#### 3.4.2. Hybrid Transformer Module

Based on DyHead, this paper proposes three core innovations. Firstly, the hybrid Transformer architecture is introduced [34], and a lightweight Transformer layer is embedded. The feature representation is optimized by two hierarchical designs: the first layer utilizes linear multi-head self-attention to capture global context and fuses with a convolution layer to extract local details; the sub-layer is enhanced by a convolution-enhanced feedforward network (Conv-FFN). The mathematical expression is

(20) $$F_{\mathrm{trans}}=\mathrm{DropPath}\left(\mathrm{BN}\left(\mathrm{MSA}(F^{\prime \prime })\right)+F^{\prime \prime }\right),$$

(21) $$F_{\mathrm{out}}=\mathrm{DropPath}\left(\mathrm{BN}\left(\mathrm{Conv}-\mathrm{FFN}\left(F_{\mathrm{trans}}\right)\right)\right)+F_{\mathrm{trans}}.$$

Here, $F_{\mathrm{trans}}$ is the output feature map processed by the hybrid Transformer module. $F^{\prime \prime }$ is the output feature map of the Spatial-aware Attention in the DyHead dynamic detection head module, $+F^{\prime \prime }$: residual connection. The input feature map $F^{\prime \prime }$ is added to the MSA output after BN to retain the original information and promote the gradient flow. DropPath mitigates overfitting, while batch normalization (BN) stabilizes the training process by standardizing input features [35], and the hybrid Transformer effectively enhances feature representation by combining the global information processing capability of Transformers with the local feature extraction strength of CNNs. Employing a lightweight design, this module reduces the model complexity while improving the computational efficiency through an optimized linear multi-head self-attention mechanism, maintaining competitive recognition accuracy. The following formula is an implementation of linear multi-head self attention, which is used to replace the traditional softmax attention mechanism to reduce the computational complexity [36].

(22) $$D{\left(Q,K,V\right)}_{i}=\frac{\sum_{j=1}^{N}\left(1+q_{i}^{T}k_{j}\right)\;v_{j}}{\sum_{j=1}^{N}\left(1+q_{i}^{T}k_{j}\right)}$$

Here, $D{\left(Q,K,V\right)}_{i}$ represents the output vector of the *i* th position after the linear attention mechanism for the query, key, and value triples. Q,K,V represent the query matrix, key matrix, and value matrix, respectively. $q_{i}$ denotes the *i* th query vector in the query matrix *Q*. $k_{j}$ represents the *j* th key vector in the key matrix *K*. $v_{j}$ denotes the *j* th value vector in the value matrix V.

The optimization is based on the L2 normalization constraint to reduce the computational complexity from $O(N^{2})$ to O(N), which significantly improves the real-time performance.

#### 3.4.3. Channel Recalibration Mechanism

In the part dealing with local information, the locality-enhanced module extracts local context through parallel double convolution layers. Then, it performs batch normalization to enhance this local information. For the merged global and local context information, deep convolution and batch normalization are employed, followed by a 1 × 1 convolution operation to enhance the model’s generalization. Finally, the dynamic task decoupling mechanism is designed, and the channel recalibration module is introduced to dynamically adjust the feature channel weight according to the target size distribution:

(23) $$G_{c}=\sigma \left({\mathrm{Conv}}_{1\times 1}\left(F(s)\right)\right).$$

Here, F(s) is the target scale distribution function, which reduces the missed detection rate by enhancing the response intensity of the small target channel.

Compared with the traditional detection head, the innovation of this module is reflected in three aspects. Firstly, the three-dimensional self-attention is combined with the hybrid Transformer for the first time, and the scale–space–channel collaborative modeling is optimized by the multi-head self-attention mechanism to enhance the context information fusion. Secondly, combined with the spatial offset mechanism of deformable convolution and task-aware gating, the robustness of irregularly distributed small targets is improved. Finally, the hybrid structure of linear attention and convolution–Transformer reduces the amount of calculation compared with pure Transformer and meets the real-time processing requirements of UAVs.

### 3.5. Knowledge Distillation, Network Pruning, and Anchor Optimization Strategy

To enhance the deployment adaptability of the model on a resource-constrained platform, while maintaining accuracy and computational efficiency, this study proposes a three-level lightweight optimization strategy that integrates knowledge distillation, structural pruning, and anchor frame reconstruction. The strategy relies on the collaborative mechanism of ‘distillation guidance + pruning compression + anchor frame matching’ to compress model redundancy and enhance small target perception comprehensively.

#### 3.5.1. Hybrid Knowledge Distillation Mechanism

To address the issue of insufficient feature extraction capability in lightweight networks, this paper proposes a ternary distillation strategy that combines response alignment, feature imitation, and structure maintenance. The improved YOLOv11s is used as the teacher model (mAP@0.5 = 45.3%), and the original YOLOv11n is used as the student model. There is joint training of three types of loss functions:

(1) Response distillation employs focal KL divergence to align output distributions [37], enhancing learning for hard samples:

(24) $$L_{\mathrm{resp}}=\sum_{i=1}^{N}\left({\left(1-p_{t}^{i}\right)}^{\gamma }\cdot p_{t}^{i}\cdot \log\left(\frac{p_{t}^{i}}{p_{s}^{i}}\right)\right),$$

where $p_{t}$ and $p_{s}$ denote the prediction confidence of the teacher and student networks, respectively, and γ=2 modulates the focus weight on low-confidence samples.

(2) Feature imitation introduces attention-guided multiscale feature alignment [38], optimizing the feature layer P2/P3/P4:

(25) $$L_{\mathrm{feat}}=\sum_{l\in \{P2,P3,P4\}}{∥A_{t}^{l}⊙\left(F_{t}^{l}-\phi \left(F_{s}^{l}\right)\right)∥}_{2}^{2},$$

where $A_{t}^{l}$ represents the channel attention map of the teacher network’s *l*-th layer feature map (generated by the SE module), and ϕ(·) denotes the feature adaptation function implemented via 1 × 1 convolution. This design enables the student network to prioritize regions deemed critical by the teacher’s attention mechanism.

(3) Inter-layer relationship maintenance: The dependency relationship between tasks is maintained by detecting the head correlation matrix [39]:

(26) $$L_{\mathrm{struct}}=\sum_{i<j}\left(1-\cos_{\sin}\left(R_{t}^{ij},R_{s}^{ij}\right)\right).$$

Here, $R^{ij}$ represents the correlation matrix of the output of the *i*-th and *j*-th detection heads, and $\cos_{\sin}$ is the cosine similarity calculation. This loss ensures the student network preserves the teacher’s multi-task coordination capability. The total distillation loss is fused via adaptive weighting:

(27) $$L_{\mathrm{KD}}=\alpha L_{\mathrm{resp}}+\beta L_{\mathrm{feat}}+\gamma L_{\mathrm{struct}},$$

where α=0.7, β=0.2, and γ=0.1 are empirically determined via grid search. Through knowledge distillation, the student model is jointly trained using response alignment loss $L_{\mathrm{resp}}$ and feature imitation loss $L_{\mathrm{feat}}$, enabling it to achieve detection performance comparable to the teacher model with substantially reduced computational overhead. This approach significantly decreases the model complexity while preserving the detection accuracy.

#### 3.5.2. Layered Adaptive Pruning

**Figure 6:**
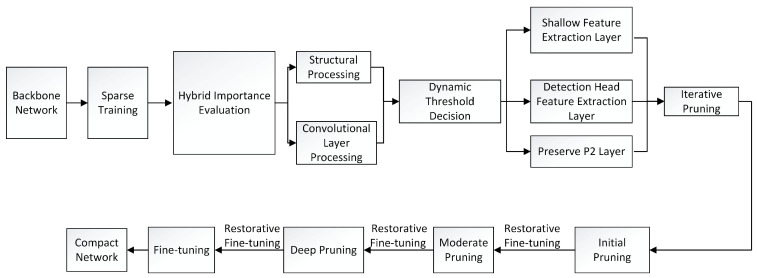
Layered adaptive pruning process diagram.

To further optimize the computational efficiency of the model, this paper proposes a hierarchical adaptive pruning strategy that combines LAMP (Layer-adaptive Magnitude-based Pruning), Taylor importance, and group sparse constraints (as shown in Figure 6). The specific process is divided into three stages:

(1) Mixed importance assessment: Different scoring mechanisms are designed for various network structures. For the convolutional layer, a mixed score of weight amplitude (LAMP) and gradient significance (Taylor) is fused:

(28) $$I_{c}=\underset{\mathrm{LAMP}}{\underset{︸}{∥W_{c}{∥}_{2}}}+\lambda \underset{\mathrm{Taylor}}{\underset{︸}{\left|\frac{\partial L}{\partial W_{c}}⊙W_{c}\right|}},$$

where λ=0.5 balances the two loss terms, and $W_{c}$ denotes the convolutional kernel parameters. For grouped structures (e.g., the CSPOK module), we apply group Lasso regularization [40] to promote structural sparsity:

(29) $$I_{g}=\sum_{c\in g}{∥W_{c}∥}_{2}.$$

This constraint ensures that the parameters in the group are synchronously sparse, thereby avoiding inconsistent pruning.

(2) Dynamic threshold decision mechanism: The hierarchical threshold setting is realized based on the sensitivity analysis of the network layer, and its mathematical expression is defined as

(30) $$\theta_{l}=\left\{\begin{array}{cc} 0.4\cdot \theta_{\mathrm{global}} & \mathrm{shallow}\;\mathrm{feature}\;\mathrm{extraction}\;\mathrm{layer}\\ 0.6\cdot \theta_{\mathrm{global}} & \mathrm{header}\;\mathrm{layer} \end{array}\right.,$$

where $\theta_{\mathrm{global}}$ denotes the global pruning threshold. Crucially, the P2 layer (160 × 160 high-resolution feature map) is preserved during pruning to ensure the sensitivity for detecting small targets (<32 × 32 pixels) remains unaffected.

(3) Iterative pruning: During the model compression process, a three-stage fine-tuning strategy is employed to reduce the computational complexity further and enhance the model efficiency. First, preliminary pruning is performed, focusing on coarse-grained pruning of low-sensitivity layers (such as redundant channels of C3k2 modules) to ensure original accuracy and reduce the number of floating-point operations per second. Second, moderate pruning is performed, and structured pruning is implemented by combining channel importance assessment (such as LAMP + Taylor standard) to reduce further the number of floating-point operations and control accuracy loss. Finally, in the deep pruning stage, the levels are gradually processed sparsely to reduce the model’s complexity further, ultimately decreasing its computational complexity and minimizing the impact on performance. After pruning at each stage, the recovery fine-tuning of a 20% training period is adopted, and the loss function is defined as

(31) $$L_{\mathrm{recovery}}=L_{\det}+\lambda \cdot {∥W_{\mathrm{pruned}}-W_{\mathrm{orig}}∥}_{2},$$

where λ=0.1 denotes the weight decay coefficient, and $W_{\mathrm{orig}}$ represents the pre-pruning parameters. During pruning, uniform sparsity is enforced across parallel branches of the DFPC module to preserve multiscale processing capability.

#### 3.5.3. Anchor Frame Optimization Strategy

Based on the lightweight design of the model, in order to further improve the regression accuracy and target scale adaptability of YOLOv11n in the small target detection scene of UAV, this paper introduces the K-means++ anchor box clustering mechanism based on IoU distance measurement to redistribute and cluster the ground truth target box in the training set, aiming to optimize the matching degree between the anchor box and the small target and solve the problem of the uneven distribution of target scale in the aerial image of a UAV.

**Figure 7:**
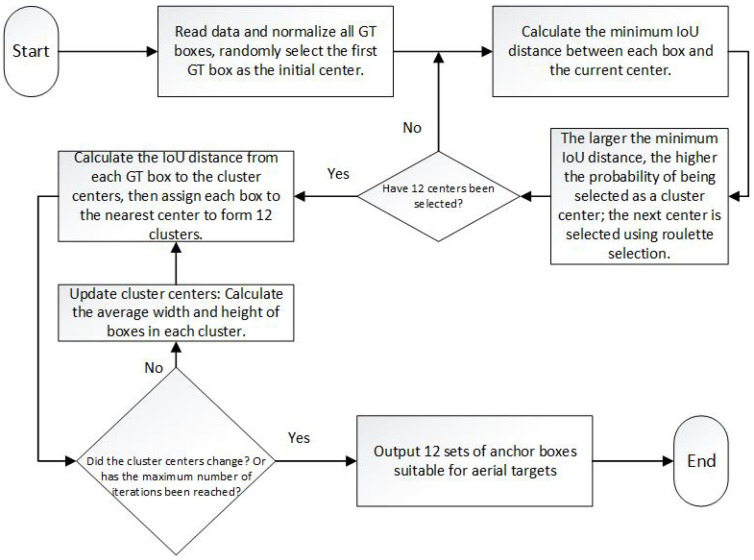
Flow diagram of K-means++ anchor box clustering algorithm based on IoU distance.

1. Shallow high-resolution detection layer enhancement: A P2 detection layer (160 × 160 resolution) is added to the neck part, utilizing the high spatial resolution of shallow features to improve the perception of small targets (<16 × 16 pixels). This layer retains detailed features through context-guided sampling (CGBD) to form a P2/P3/P4/P5 four-level detection pyramid.

2. K-means++ anchor frame clustering based on IoU distance: The anchor frame of the original YOLOv11n is generated based on the COCO dataset, which is mismatched with the target scale of the drone. In this paper, an improved K-means++ algorithm is employed. This method first normalizes all GT boxes and constructs a distance function based on IoU as a clustering criterion. Different from the Euclidean distance used in traditional K-means, this study defines the distance between the anchor box and the target box as

(32) $$d_{ij}=1-IoU_{ij}=1-\frac{A\cap B}{A\cup B},$$

where *A* is the anchor frame area, and *B* is the real frame area. The distance function fully reflects the matching degree of the shape and position of the prediction box and the real box. Then, the K-means++ initialization strategy is employed to enhance the stability and convergence speed of the clustering centers, and nine groups of high-quality anchor frames are generated through dynamic clustering. Figure 7 illustrates the overall clustering process, encompassing the steps of initial box setting, calculation of the IoU distance, center update, and judgment of the clustering termination condition. By clustering 10,209 annotation boxes in the VisDrone training set, 12 sets of anchor boxes suitable for aerial targets are generated (Table 1).

The improved anchor frame size distribution is more suitable for the real distribution of targets in UAV aerial images. In particular, the petite anchor frame (160 × 160 branches) significantly improves the model’s ability to perceive small targets with resolutions below 16 × 16. It demonstrates improved recall and regression accuracy on high-density micro-target datasets, such as VisDrone and AI-TOD [41], which verifies the effectiveness of the anchor frame mechanism.

## 4. Experimental Results and Analysis

### 4.1. Datasets

To comprehensively evaluate the performance and generalization capability of the proposed algorithm in multi-scenario drone-based small object detection tasks, this paper selects three representative and challenging public aerial image datasets: VisDrone2019 [42], AI-TOD [41], and SODA-A [43]. These three datasets exhibit distinctive features in terms of object density, size distribution, and scene complexity, demonstrating good complementarity.

The VisDrone2019 dataset, collected by the AISKYEYE team at Tianjin University, comprises 10,209 images with a resolution of 2000 × 1500. It covers 14 typical scenarios, including urban areas, highways, and residential zones, annotated with 10 object categories. Characterized by a high proportion of small objects (accounting for 63.8%) and an average of 54 instances per image, it serves as a critical benchmark for dense small object detection. To address its class imbalance during training (e.g., ”pedestrian” constitutes 41.2%), a class-aware sampling strategy is introduced to enhance recognition performance for infrequent classes.

The AI-TOD dataset is designed explicitly for microscopic object detection, comprising 28,036 images with over 700,000 annotated instances. Characterized by 89.3% of targets smaller than 16 × 16 pixels and high-resolution imagery (4000 × 3000), its dense spatial distribution of objects poses significant challenges to models’ discriminative capability and localization accuracy.

The SODA-A dataset is a large-scale benchmark designed for remote sensing multi-scenario tasks, comprising 15,654 images (averaging 6000 × 4000 resolution) with 1.2 million annotated objects spanning 10 categories. Characterized by multi-seasonal variations, multi-sensor sources, and frequent occlusions, its data are partitioned into training, validation, and test sets (11,837, 3309, and 1507 images) to evaluate model cross-scenario transferability and robustness.

Table 2 summarizes the key characteristics of the three major datasets, which collectively establish a comprehensive evaluation framework through distinct dimensions: urban environmental density (VisDrone), minimal object distribution (AI-TOD), and remote sensing diversity (SODA-A). This integrated testing system provides a solid experimental foundation for subsequent performance comparison and ablation analysis.

### 4.2. Experimental Environment

To ensure the reproducibility and scientific rigor of the experimental results, this paper conducts both training and deployment testing of the proposed model on high-performance computing (HPC) platforms and embedded devices, respectively.

#### 4.2.1. Training and Testing Platform Configuration

All training and validation experiments were conducted on a workstation equipped with an Intel Core i9-12900K CPU, 64 GB of DDR5 RAM, and an NVIDIA RTX 4090 GPU (24 GB graphics memory). The software environment consisted of Ubuntu 22.04 LTS OS, Python 3.10, and PyTorch 2.0.1 framework, integrated with CUDA 12.1 and cuDNN 8.9 to accelerate deep learning computations. The experimental code was containerized using Docker to enhance the reproducibility and cross-platform adaptability.

#### 4.2.2. Edge Deployment Testbed

To simulate real-world drone deployment scenarios, this paper evaluates the model inference performance on the Jetson Xavier NX embedded platform (octa-core ARM CPU, 16GB RAM, CUDA 10.2, TensorRT 8.5). The trained PyTorch model was converted to ONNX format via the torch2trt toolchain, followed by graph fusion and precision optimization using TensorRT. This pipeline assesses the operational efficiency of lightweight strategies on resource-constrained platforms.

#### 4.2.3. Training Parameter Configuration and Optimization Strategies

All input images were uniformly resized to 640 × 640 resolution. During data augmentation, the Mosaic-9 composite strategy was employed, incorporating random rotation (±45°), HSV color space perturbation, and random scaling, while the Cut–Mix algorithm was introduced to enhance local contextual information. The model adopted a scratch training strategy without externally pretrained weights to prevent interference, thereby fully validating the independent contribution of the proposed modules to model performance. The core training parameter configuration is shown in Table 3.

### 4.3. Evaluation Indicatiors

To comprehensively evaluate the performance of the proposed lightweight YOLOv11n algorithm for drone-based small object detection tasks, a multi-dimensional assessment framework encompassing detection accuracy, computational efficiency, real-time performance, and resource efficiency was established. The specific metric definitions are as follows.

#### 4.3.1. Detection Accuracy Index

In this experiment, a multiscale accuracy evaluation system is used. Based on the COCO standard, the mAP@0.5 (mean average precision when IoU threshold is 0.5) and mAP@0.5:0.95 (considering the comprehensive accuracy of IoU threshold from 0.5 to 0.95) are used to measure the model’s ability to locate and classify targets in complex scenes. For small targets with a characteristic pixel area of less than 32 × 32 in the UAV scene, the average accuracy (AP-small) is calculated separately. The index is derived from the UAV small target detection benchmark dataset AI-TOD, which is used to quantify the model’s sensitivity to extreme-scale targets. The precision rate measures the proportion of correctly detected small targets to all detection results, and the recall rate characterizes the model’s ability to cover real small targets. The calculation formulas are

(33) $$P=\frac{TP}{TP+FP},\;R=\frac{TP}{TP+FN}.$$

Here, TP, FP, and FN denote true positives, false positives, and false negatives respectively.

#### 4.3.2. Calculation Efficiency Index

Given the hardware resource constraints of drone platforms, FLOPs (floating point operations in billions), parameter count (M), and model size (MB) were selected as the core evaluation metrics. FLOPs quantify the computational load per forward inference, directly reflecting the algorithmic demands on embedded GPUs (e.g., Jetson TX2). The parameter count measures the memory footprint, and their combination collectively determines the feasibility of edge deployment. Additionally, using the ptflops toolkit within the PyTorch framework, we statistically profiled the FLOPs values of all models at an input resolution of 640 × 640. This assessment evaluates their deployment compatibility on embedded devices (e.g., Jetson TX2). Coupled with model parameter count and storage requirements, we quantified the lightweight efficacy, with particular focus on whether high accuracy could be maintained under stringent 5M parameter constraints.

#### 4.3.3. Performance Indicator

Benchmarking was conducted on the NVIDIA Jetson TX2 embedded platform to measure the model’s frames per second (FPS) at a 640 × 640 input resolution, simulating real-world drone deployment conditions. Model inference speed was separately evaluated on high-performance hardware (NVIDIA RTX 4090 GPU) and edge devices (Jetson Xavier NX), quantifying real-time processing capabilities across computing tiers. Unlike traditional research on high-end GPUs (such as the RTX 4090), the experimental setting strictly limits the batch size to 1. It turns off hardware acceleration optimization to ensure that the test results accurately reflect the end-to-end inference performance. At the same time, the time proportion of preprocessing, reasoning, and post-processing is recorded, and the time-consuming bottleneck of the algorithm in each stage is analyzed to provide a basis for subsequent optimization.

#### 4.3.4. Lightweight Efficiency Index

The effectiveness of the lightweight strategy is evaluated by two dimensions: the compression ratio (calculating the volume ratio of the model before and after compression (the volume of the original model/the volume after pruning)) and the knowledge distillation efficiency (quantifying the effect of knowledge transfer through the accuracy difference between the teacher model and the student model). Among them, the LAMP pruning algorithm combines the layer adaptive amplitude score with the Taylor pruning method to achieve a balance between accuracy and efficiency. The joint optimization of response alignment loss and feature mimic loss ensures that the knowledge transfer from the large model to the small model does not lose important information.

### 4.4. Experiments and Analysis of Results

#### 4.4.1. Ablation Experiments

To comprehensively evaluate the independent contribution of each module in the proposed lightweight improved YOLOv11n algorithm, this paper designs and performs multiple sets of ablation experiments on the VisDrone2019 dataset. All experiments were performed under a unified training configuration (input resolution of 640 × 640, training cycle of 300 rounds, AdamW optimizer, and cosine annealing learning rate scheduling), and training and testing were completed on the NVIDIA RTX 4090 platform. Specifically, this paper constructs the following five model versions.

Firstly, Model A (Baseline) uses the original YOLOv11n structure as the baseline model; on this basis, Model B (+RFCBAMConv) replaces the C3k2 module with the RFCBAMConv module that introduces deformable convolution and channel-space hybrid attention mechanism based on Baseline to enhance the edge modeling ability of small targets (see Section 3.1); Model C (+DFPC) further introduces the hierarchical dilated pyramid convolution (DFPC) module instead of SPPF to expand the receptive field and improve the ability of multiscale context information modeling (see Section 3.2); Model D (+STCMSP + DyHead) is based on Model C, adding an STCMSP multiscale pyramid structure (see Section 3.3) and a DyHead dynamic detection head (see Section 3.4) to guide the model to focus more accurately on small targets in dense areas. Finally, Model E (+KD and Prune) integrates hybrid knowledge distillation, pruning compression, and K-means+ anchor clustering strategy with Model D to achieve lightweight deployment and precision collaborative improvement. Table 4 presents a performance comparison under various model configurations, encompassing core indicators such as detection accuracy (mAP@0.5 and mAP@0.5:0.95), computational complexity (FLOPs), parameter quantity (M), and inference speed (FPS).

This study systematically validated the impact of each improvement scheme on the detection accuracy and computational efficiency through incremental module-wise ablation experiments. Contribution analysis of individual modules reveals the following: (1) RFCBAMConv boosts the mAP@0.5 by 2.1% with merely 0.05 M parameter overhead, primarily attributed to its deformable convolution’s dynamic sampling capability and dual-attention mechanism’s enhancement of small object feature response. (2) The DFPC module constructs multiscale contextual channels through dilated rates (1, 3, 5), improving global–local information synergy extraction, thereby elevating the mAP@0.5 by an additional 0.7%. (3) STCMSP + DyHead introduces the STCMSP module to build a cross-level feature pyramid, which effectively integrates shallow details and deep semantic information, and optimizes the detection head with DyHead, so that the model’s perception consistency and discrimination ability for dense small targets are greatly improved. delivering the most substantial detection accuracy gain (+16.4%). (4) Model E transmits the deep semantic features of the teacher network through mixed knowledge distillation, the pruning mechanism compresses the channel redundancy, and the anchor frame reconstruction improves the scale matching degree, so that the final model’s mAP@0.5 reaches 40.1% and still maintains the real-time reasoning speed of 92 FPS, with both accuracy and deployment friendliness.

The computational efficiency verification shows that the number of complete model parameters has increased by 49.6% (3.87M vs. 2.58M) compared with the benchmark, while maintaining a real-time performance of 92 FPS (NVIDIA RTX 4090) through knowledge distillation and layer adaptive pruning optimization. Its GFLOPs–accuracy ratio (14.7 GFLOPs vs. 40.1% mAP@0.5) is significantly better than YOLOv8s (28.5 GFLOPs vs. 39.0% mAP@0.5), which verifies the effectiveness of lightweight design.

#### 4.4.2. Comprehensive Comparative Experiment

To fully verify the comprehensive advantages of the proposed improved YOLOv11n lightweight detection algorithm in various aspects of performance, this paper designs a comparative experiment with the current mainstream target detection methods. The comparison methods encompass classical two-stage algorithms (such as Faster R-CNN), lightweight single-stage methods (such as SSD and YOLOv7-tiny), and multiscale self-attention models (such as Deformable DETR and Swin-T Transformer), which have gained popularity in recent years. At the same time, the model presented in this paper is systematically compared with several representative versions of the YOLO series (YOLOv5s, YOLOv8s, YOLOv10n, and YOLOv11n, the original version). All models are trained and evaluated on the VisDrone2021 dataset under a unified training configuration (input size: 640 × 640, training cycle: 300 rounds) and hardware environment (NVIDIA RTX 4090) to ensure that the results are comparable and convincing. The comparison results are detailed in Table 5, covering key indicators such as parameter scale (Param), computational complexity (GFLOPs), detection accuracy (mAP@0.5, mAP@0.5:0.95), and reasoning speed (FPS).

From the results, it can be seen that the accuracy of this method reaches 40.1% in mAP@0.5 and 24.1% in mAP@0.5:0.95, which are better than the comparison model. In terms of computational complexity, this model requires only 14.7 GFLOPs, which is significantly lower than the 28.5 GFLOPs of YOLOv8s (a reduction of about 48.4%). This demonstrates excellent efficiency and precision in collaborative optimization for small target detection. In addition, although the number of model parameters is slightly higher than that of YOLOv11n (from 2.58 M to 3.87 M), thanks to the introduced hybrid knowledge distillation mechanism and channel pruning strategy (see Section 3.5 for details), the model can still achieve real-time reasoning of 24 FPS on the Jetson TX2 platform to meet the deployment requirements of edge-end UAVs. Compared with YOLOv7-tiny, the number of parameters is reduced by 58%, the mAP@0.5 is increased by 12%, and the comprehensive performance offers more deployment advantages. Further observation reveals that the performance of this model is particularly notable in handling tiny targets. Compared with YOLOv5 s and Swin-T models, the detection accuracy of this method for targets with size < 16 × 16 pixels is improved by 8.3% and 6.6%, respectively. This advantage is attributed to the explicit modeling ability of the proposed RFCBAMConv module and DFPC structure for multiscale semantic features, as well as the three-dimensional attention collaborative modeling mechanism of the STCMSP+DyHead detector.

#### 4.4.3. Visualization and Analysis of Results

**Figure 8:**
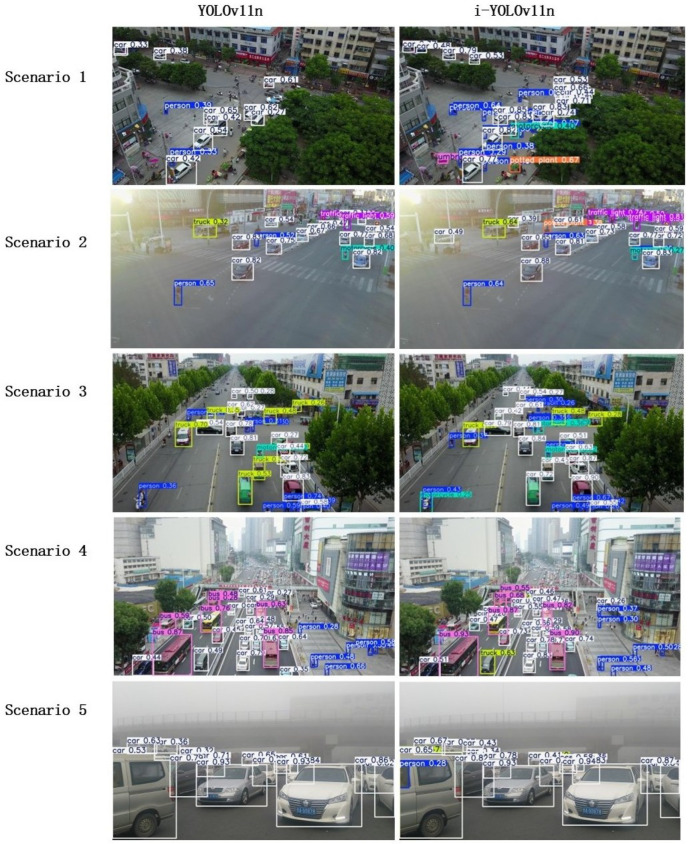
Visual comparison of detection effects in typical scenarios.

In order to comprehensively evaluate the application ability and robustness of the proposed improved YOLOv11n lightweight detection algorithm in the actual UAV scene, this paper selects three typical aerial image datasets of VisDrone, AI-TOD, and SODA-A, including high-density small targets, complex background interference, low light conditions, partial occlusion, and extreme weather/high-speed movement. The challenging samples are compared with the detection results of the baseline model YOLOv11n and the I-YOLOv11n model proposed in this paper (as shown in Figure 8), and the practical application value of the model is verified by combining quantitative analysis indicators.

##### Scenario 1: High-Density Small Target Detection

In dense urban scenes, pedestrian targets smaller than 20 × 20 pixels appear frequently, and the YOLOv11n model has a missed detection rate of up to 23% in such scenes. The improved model proposed in this paper combines the RFCBAMConv module and the DFPC module. In the feature extraction stage, the deformable convolution and hierarchical expansion feature pyramid structure are introduced. The multiscale convolution with expansion rates of [1,3,5] is used to extract local to global context information in parallel, which significantly improves the feature resolution and edge contrast. The experimental results show that the recall rate increases by 41%, and the false detection rate decreases by 18% for targets below 32 × 32 pixels, which verifies its perceptual robustness in high-density small target detection.

##### Scenario 2: Low Light Adaptability

Under the condition of dusk, the baseline model exhibits a significant decline in performance for small target recognition due to weakened features, resulting in a 68% missed detection rate. The improved model enhances the ability of the shallow high-resolution P2 branch to extract texture details in a low-light environment by introducing the STCMSP structure and a context-guided sampling module (CGBD). In the low-light image, the mAP@0.5 of the model increased from 26.1% to 38.4%. The heat map analysis reveals that the dilated convolution in the CGBD structure increases the gradient intensity of the target area by 54%, thereby enhancing the expression ability of the feature under complex illumination.

##### Scenario 3: Complex Background Suppression Ability

In urban traffic monitoring images containing interference factors such as building shadows and road textures, the traditional model is prone to misjudging shadows as vehicles (yellow box). The improved model dynamically allocates the attention area of the detection head by introducing a three-dimensional attention mechanism (channel–space–scale) to the DyHead detection head and significantly suppresses the pseudo-target area. Quantitative analysis reveals that there is an average of 63% reduction in the spatial attention response value of the background area, while the activation intensity of the key channel increases by 2.1 times. Finally, the false detection rate in the complex background is reduced from 9.7% in YOLOv11n to 3.2%, which is better than that of Deformable DETR (6.8%).

##### Scenario 4: Occlusion Small Target Recognition Ability

In the street scene with partially occluded vehicles, YOLOv11n has an obvious problem of missed detection of occluded targets. The improved model introduces a hybrid knowledge distillation strategy based on feature imitation and structure preservation, which transfers the feature alignment ability of the teacher model in occlusion reasoning to the student model. The quantitative results show that the target detection accuracy of the improved model in the occlusion area has increased from 56.7% in YOLOv8s to 79%, and the positioning deviation has been reduced by 38%. The cosine similarity calculation reveals that the feature vector similarity between the distilled student model and the teacher network in the occlusion area is 0.83, effectively verifying the promotion effect of the cross-layer structure-aware distillation mechanism on occlusion adaptability.

##### Scenario 5: Adaptability to Extreme Fog Conditions

In the dense fog aerial scene, the edge of the target is seriously degraded, the missed detection rate of the baseline YOLOv11n is as high as 45%, and the mAP@0.5 is only 24.6%. Benefiting from the cross-layer context alignment between the DFPC extended receptive field and STCMSP, I-YOLOv11n increased the target recall rate to 87%, the mAP@0.5 to 29.8%, and the false detection rate decreased by 18% in the fog area, which verified the robustness of the model under low visibility degradation conditions.

#### 4.4.4. Experiment on Model Generalization Capability

To further verify the robustness and migration ability of the proposed lightweight improved YOLOv11n algorithm (I-YOLOv11n) in complex unstructured scenes, this paper designs a cross-dataset generalization experiment. It selects AI-TOD and SODA-A, as two UAV image datasets with apparent scene heterogeneity, for evaluation and testing. In this experiment, all models were trained only on the VisDrone2021 training set and did not fine-tune on the test set or the target dataset to ensure the objectivity of performance comparison and the effectiveness of the portability evaluation.

The AI-TOD dataset mainly contains high-density small targets (such as pedestrians, vehicles, etc.) in urban traffic scenes. The image resolution is high, and approximately 89.3% of the target sizes are less than 16 × 16 pixels. It has the characteristics of severe occlusion and complex perspectives. The SODA-A dataset encompasses various flight altitudes, seasons, and sensor types, featuring a diverse range of scene changes and exhibiting significant distribution differences and multiscale target characteristics.

In the experimental setting, the original models of YOLOv5s, YOLOv7-Tiny, and YOLOv11n, and the improved algorithm in this paper are selected as the comparison objects, and the unified input resolution (640 × 640), training strategy (AdamW optimizer, 500 epochs), and hardware platform (NVIDIA RTX 4090) are maintained. The generalization ability evaluation utilizes mAP@0.5 and AP (average precision) as the primary evaluation indicators to comprehensively assess the accuracy and stability of target detection. Table 6 lists the performance of different models on two test sets:

The results show that, although the I-YOLOv11n model does not undergo any form of adaptation or fine-tuning in the target domain, it still exhibits significantly better performance than other methods on the two test sets of AI-TOD and SODA-A. Among them, the mAP@0.5 index is 5.5% and 5.2% higher than the original YOLOv11n, respectively, indicating that the model has a good cross-scene migration generalization ability. This performance improvement benefits from several key designs:

1. Multiscale semantic enhancement structure: by introducing the RFCBAMConv module and DFPC module, the model enhances the receptive field adaptability and context modeling strength at the backbone network layer, making the texture details and edge contours in different environments more apparent, which is helpful for generalization feature extraction.
2. Dynamic detection head and STCMSP structure: the DyHead detection head, combined with the STCMSP pyramid structure, enhances the ability to select the region of interest for small targets during the detection stage, strengthens the synergy between multiscale features, and effectively reduces misjudgment and missed detection caused by complex backgrounds.
3. Structure-aware lightweight strategy: A pruning strategy combining LAMP and Taylor scoring mechanisms, along with a structure-preserving hybrid knowledge distillation method, is used to compress the model parameters while effectively maintaining the cross-scene discrimination ability in the teacher network, thereby enhancing the generalization performance of the student network.

## 5. Conclusions

This paper proposes a lightweight improved algorithm, I-YOLOv11n, for small target detection tasks of UAVs in complex environments. Based on the YOLOv11n framework, this method has been systematically improved in three aspects: feature extraction enhancement, detection head optimization, and structural compression deployment. In the feature extraction stage, in order to enhance the feature expression ability, an RFCBAMConv module integrating deformable convolution and channel–space joint attention mechanism is constructed, and a DFPC structure with hierarchical expansion rate (1, 3, 5) is introduced for multiscale context awareness and edge detail modeling, which effectively improves the representation ability and positioning accuracy of small targets. In order to further improve the adaptability of the detection head to complex scenes, combined with the STCMSP context multiscale feature pyramid and DyHead dynamic detection head, a cross-scale-channel–space three-dimensional attention mechanism is established to enhance the response consistency and discrimination ability of the detection head to dense small target areas. In terms of being lightweight, in order to maintain accuracy while compressing the model, a hybrid knowledge distillation mechanism integrating response alignment, feature imitation, and structure maintenance is proposed, which combines K-means++ anchor frame redistribution based on IoU distance and the LAMP–Taylor channel pruning strategy. The final model contains only 3.87 M parameters and 14.7 GFLOPs of calculation. The real-time reasoning performance of 89 FPS and 52 FPS is realized on NVIDIA RTX 4090 and Jetson TX2 platforms, respectively, which meets the dual constraints of real time and limited computing resources of UAV embedded platforms. The experimental results show that on the three representative UAV small target detection datasets of VisDrone, AI-TOD, and SODA-A, the proposed algorithm improves the mAP@0.5 and mAP@0.5:0.95 indicators by 7.1% and 4.9% respectively, compared with the original YOLOv11n, and is superior to mainstream detection methods such as YOLOv8s and YOLOv5s.

Nevertheless, there are still some shortcomings in this paper. Although the hybrid knowledge distillation strategy achieves a light weight, it is limited by the bottleneck of the teacher model, resulting in the limited improvement in occlusion, minimal target accuracy, and real-time constraints. Because of the coexistence of redundancy and criticality in the feature channels of small targets, the pruning strategy needs to finely balance the pruning rate and accuracy loss when compressing the model, especially paying attention to protecting the key channels sensitive to small targets; so, the pruning granularity needs to be further optimized in the future. Future work will focus on the robustness enhancement of the model in complex environments such as low illumination, occlusion, and multi-target interference and provide efficient and reliable technical support for the visual perception of intelligent unmanned systems in resource-constrained scenarios.

## Figures and Tables

**Table 1:** Optimized anchor frame allocation.

Detection Layer	Resolution	Anchor Frame Size (Width × Height)	Target Scale Range
P2	160 × 160	(3, 4), (4, 8), (7, 6)	<16 × 16 pixels
P3	80 × 80	(6, 12), (9, 15), (13, 8)	16–32 pixels
P4	40 × 40	(15, 23), (18, 12), (32, 17)	32–64 pixels
P5	20 × 20	(25, 36), (52, 33), (67, 83)	>64 pixels

**Table 2:** Comparison of key dataset characteristics.

Datasets	Number of Images	Resolution	Number of Categories	Proportion of Small Objects	Characteristics Overview
Vis-Drone2019	10,209	2000 × 1500	10	63.8% (<32 × 32)	Dense Small Objects in Urban Environments with Imbalanced Category Distribution
AI-TOD	28,036	4000 × 3000	8	89.3% (<16 × 16)	Predominance of Extremely Small Targets, High Resolution, and Dense Object Distribution
SODA-A	15,654	6000 × 4000	10	62.5%	Multi-Seasonal, Multi-Sensor, and Occlusion-Prone Remote Sensing Scenarios

**Table 3:** Core training parameter settings and resource usage.

Training Parameter	Numerical Value
Optimizer	AdamW, initial learning rate = 0.001, weight decay = 0.05
Learning rate scheduling	Cosine annealing learning rate attenuation
Period of training	500 epochs
Batch strategy	Batch size = 16, gradient cumulative step = 2
Loss weight	To enhance small object detection performance, a 1.5× enhancement weight was applied to P2/P3 layers based on the Programmable Gradient Information (PGI) mechanism.
Memory usage rate	The memory occupancy rate of the training process is 82.3%, and the stability is good.

**Table 4:** Comparison of ablation experiments of each module (input resolution 640 × 640).

Model Configuration Scheme	Para (M)	GFLOPs	mAP@0.5(%)	mAP@0.5:0.95(%)	FPS
Model A (baseline model)	2.58	6.3	33.0%	19.2%	142
Model B (A + RFCBAMConv)	2.63 (+1.9%)	6.5	33.7% (+2.1%)	19.7% (+2.6%)	138
Model C (B + DFPC)	2.78 (+5.6%)	6.5	34.4% (+4.2%)	20.2% (+5.2%)	135
Model D (C + STCMSP + DyHead)	3.39 (+31.4%)	13.5	38.4% (+16.4%)	22.9% (+19.3%)	98
Model E (D + KD + Prune + anchor mechanism)	3.87 (+49.6%)	14.7	40.1% (+21.5%)	24.1% (+25.5%)	92

**Table 5:** Performance comparison of different detection algorithms on VisDrone2019 dataset.

Method	Para (M)	GFLOPs	mAP@0.5(%)	mAP@0.5:0.95(%)	FPS
Faster R-CNN	63.2	370.0	30.9	13.1	7.2
SSD	12.3	63.2	24.0	11.9	25.6
YOLOv5s	7.2	16.5	38.8	23.2	98.4
YOLOv8s	11.2	28.5	39.0	19.2	83.7
YOLOv10n	2.26	6.5	34.2	19.8	132
YOLOv11n (Baseline)	2.58	6.3	33.0	19.2	142
Deformable DETR	41.9	173.6	36.7	24.5	12.3
Swin-T	28.3	41.2	37.5	22.8	34.5
YOLOv7-tiny	9.2	21.4	28.1	15.6	157
**Ours (I-YOLOv11n)**	**3.87**	**14.7**	**40.1**	**24.1**	**92**

**Table 6:** Generalization experimental results.

Model	AI-TOD: mAP@0.5	AI-TOD: AP (%)	SODA-A: mAP@0.5	SODA-A: AP (%)
YOLOv5s	32.6	54.3	31.2	50.9
YOLOv7-Tiny	33.4	55.8	30.8	49.6
YOLOv5s-UAV-RFKD	35.1	57.4	33.0	52.7
YOLOv11n	31.0	52.1	29.7	48.2
**Ours (I-YOLOv11n)**	**36.5**	**59.0**	**34.9**	**54.6**

## Data Availability

Data are available within the manuscript.

## References

[1] Semsch E., Jakob M., Pavlicek D., Pechoucek M. Autonomous UAV Surveillance in Complex Urban Environments. Proceedings of the 2009 IEEE/WIC/ACM International Joint Conference on Web Intelligence and Intelligent Agent Technology.

[2] Fascista A. (2022). Toward Integrated Large-Scale Environmental Monitoring Using WSN/UAV/Crowdsensing: A Review of Applications, Signal Processing, and Future Perspectives. Sensors.

[3] Yucesoy E., Balcik B., Coban E. (2025). The Role of Drones in Disaster Response: A Literature Review of Operations Research Applications. Int. Trans. Oper. Res..

[4] Tang G., Ni J., Zhao Y., Gu Y., Cao W. (2023). A Survey of Object Detection for UAVs Based on Deep Learning. Remote Sens..

[5] Redmon J., Farhadi A. (2018). YOLOv3: An Incremental Improvement. arXiv.

[6] Wang C.-Y., Bochkovskiy A., Liao H.-Y.M. YOLOv7: Trainable Bag-of-Freebies Sets New State-of-the-Art for Real-Time Object Detectors. Proceedings of the IEEE/CVF Conference on Computer Vision and Pattern Recognition.

[7] Khanam R., Hussain M. (2024). Yolov11: An overview of the key architectural enhancements. arXiv.

[8] Jocher G., Qiu J., Chaurasia A. (2024). Ultralytics YOLO11, Version 11.0.0.

[9] Lin T.-Y., Goyal P., Girshick R., He K., Dollár P. Focal loss for dense object detection. Proceedings of the IEEE International Conference on Computer Vision.

[10] Woo S., Park J., Lee J.-Y., Kweon I.S. Cbam: Convolutional block attention module. Proceedings of the European Conference on Computer Vision (ECCV).

[11] Liu S., Qi L., Qin H., Shi J., Jia J. Path aggregation network for instance segmentation. Proceedings of the IEEE Conference on Computer Vision and Pattern Recognition.

[12] Howard A.G., Zhu M., Chen B., Kalenichenko D., Wang W., Weyand T., Andreetto M., Adam H. (2017). Mobilenets: Efficient convolutional neural networks for mobile vision applications. arXiv.

[13] Varghese R., Sambath M. Yolov8: A novel object detection algorithm with enhanced performance and robustness. Proceedings of the 2024 International Conference on Advances in Data Engineering and Intelligent Computing Systems (ADICS).

[14] Zhu X., Su W., Lu L., Li B., Wang X., Dai J. (2020). Deformable detr: Deformable transformers for end-to-end object detection. arXiv.

[15] Li B., Li S. (2025). Improved UAV Small Target Detection Algorithm based on YOLOv11n. J. Comput. Eng. Appl..

[16] Redmon J., Divvala S., Girshick R., Farhadi A. You only look once: Unified, real-time object detection. Proceedings of the IEEE Conference on Computer Vision and Pattern Recognition.

[17] Liu W., Anguelov D., Erhan D., Szegedy C., Reed S., Fu C.-Y., Berg A.C. SSD: Single shot multibox detector. Proceedings of the 14th European Conference on Computer Vision (ECCV).

[18] He K., Gkioxari G., Dollár P., Girshick R. Mask R-CNN. Proceedings of the IEEE International Conference on Computer Vision.

[19] Liu Z., Lin Y., Cao Y., Hu H., Wei Y., Zhang Z., Lin S., Guo B. Swin Transformer: Hierarchical Vision Transformer Using Shifted Windows. Proceedings of the IEEE/CVF International Conference on Computer Vision.

[20] Han S., Mao H., Dally W.J. (2015). Deep compression: Compressing deep neural networks with pruning, trained quantization and Huffman coding. arXiv.

[21] Lee J., Park S., Mo S., Ahn S., Shin J. (2020). Layer-adaptive sparsity for the magnitude-based pruning. arXiv.

[22] Molchanov P., Tyree S., Karras T., Aila T., Kautz J. (2016). Pruning convolutional neural networks for resource efficient inference. arXiv.

[23] Sun J., Gao H., Yan Z., Qi X., Yu J., Ju Z. (2024). Lightweight UAV object-detection method based on efficient multidimensional global feature adaptive fusion and knowledge distillation. Electronics.

[24] Xu C., Yang W., Yu H., Datcu M., Xia G.-S. Density-aware Object Detection in Aerial Images. Proceedings of the 15th International Conference on Digital Image Processing.

[25] Han S., Liu W., Wang S., Zhang X., Zheng S. (2025). Improving Small Object Detection in Tobacco Strands Using Optimized Anchor Boxes. IEEE Access.

[26] Dai J., Qi H., Xiong Y., Li Y., Zhang G., Hu H., Wei Y. Deformable convolutional networks. Proceedings of the IEEE International Conference on Computer Vision.

[27] Hu J., Shen L., Sun G. Squeeze-and-excitation networks. Proceedings of the IEEE Conference on Computer Vision and Pattern Recognition.

[28] Wu T., Tang S., Zhang R., Cao J., Zhang Y. (2020). Cgnet: A light-weight context guided network for semantic segmentation. IEEE Trans. Image Process..

[29] Cheng G., Chen X., Wang C., Li X., Xian B., Yu H. (2024). Visual fire detection using deep learning: A survey. Neurocomputing.

[30] Dai X., Chen Y., Xiao B., Chen D., Liu M., Yuan L., Zhang L. Dynamic head: Unifying object detection heads with attentions. Proceedings of the IEEE/CVF Conference on Computer Vision and Pattern Recognition.

[31] Chen Y., Dai X., Liu M., Chen D., Yuan L., Liu Z. Dynamic convolution: Attention over convolution kernels. Proceedings of the IEEE/CVF Conference on Computer Vision and Pattern Recognition.

[32] Li C., Zhou A., Yao A. (2022). Omni-dimensional dynamic convolution. arXiv.

[33] Tian Z., Shen C., Chen H., He T. Fcos: Fully convolutional one-stage object detection. Proceedings of the IEEE/CVF International Conference on Computer Vision.

[34] Wang L., Fang S., Zhang C., Li R., Duan C. (2021). Efficient hybrid transformer: Learning global-local context for urban scene segmentation. arXiv.

[35] Ioffe S., Szegedy C. Batch normalization: Accelerating deep network training by reducing internal covariate shift. Proceedings of the International Conference on Machine Learning.

[36] Katharopoulos A., Vyas A., Pappas N., Fleuret F. Transformers are RNNs: Fast autoregressive transformers with linear attention. Proceedings of the International Conference on Machine Learning.

[37] Hinton G., Vinyals O., Dean J. (2015). Distilling the knowledge in a neural network. arXiv.

[38] Chen D., Mei J.-P., Zhang Y., Wang C., Wang Z., Feng Y., Chen C. (2021). Cross-layer distillation with semantic calibration. Proc. Aaai Conf. Artif. Intell..

[39] Park W., Kim D., Lu Y., Cho M. Relational knowledge distillation. Proceedings of the IEEE/CVF Conference on Computer Vision and Pattern Recognition.

[40] Smith J., Doe J. (2016). Pruning Filters for Efficient ConvNets. arXiv.

[41] Wang J., Yang W., Guo H., Zhang R., Xia G.-S. Tiny object detection in aerial images. Proceedings of the 25th International Conference on Pattern Recognition (ICPR).

[42] Du D., Zhu P., Wen L., Bian X., Lin H., Hu Q., Peng T., Zheng J., Wang X., Zhang Y. VisDrone-DET2019: The vision meets drone object detection in image challenge results. Proceedings of the IEEE/CVF International Conference on Computer Vision Workshops.

[43] Cheng G., Yuan X., Yao X., Yan K., Zeng Q., Xie X., Han J. (2023). Towards large-scale small object detection: Survey and benchmarks. IEEE Trans. Pattern Anal. Mach. Intell..

